# An intramural uterine fibroid became submucosal in the puerperium – proposed probable mechanism: a case report

**DOI:** 10.1186/s13256-018-1624-0

**Published:** 2018-04-01

**Authors:** Elie Nkwabong

**Affiliations:** Department of Obstetrics & Gynecology, University Teaching Hospital/Faculty of Medicine and Biomedical Sciences, P.O. Box 1364, Yaoundé, Cameroon

**Keywords:** Puerperium, Large uterine fibroid, Vaginal prolapse, Urinary retention

## Abstract

**Background:**

Vaginal prolapse of a large uterine fibroid is a rare phenomenon in a woman who delivered vaginally recently, given that this fibroid might have obstructed labor. The author presents a case report of a vaginally prolapsed large pedunculated submucosal uterine myoma in a woman with a recent uncomplicated vaginal delivery.

**Case presentation:**

A 25-year-old black African woman had four intramural uterine fibroids of diameters 62 to 94 mm diagnosed in April 2013 with standard ultrasound scan. She got pregnant in July 2014. An ultrasound scan done on 31 August 2014 at 10 weeks’ gestation identified four intramural uterine fibroids, with sizes varying from 70 to 150 mm. Her pregnancy was well followed up, without any complications. She had an uneventful vaginal delivery on 10 April 2015. During uterine exploration, indicated for retention of parts of fetal membranes, no pedunculated submucosal fibroid was found. On 15 May 2015, she consulted for difficult micturition and partial urinary retention that occurred 2 days ago. A vaginally prolapsed 10 cm uterine fibroid was diagnosed. Forty-eight hours after administration of intravenously administered broad spectrum antibiotics, the myoma was successfully twisted off by means of vaginal route under general anesthesia, which relieved her symptoms.

**Conclusions:**

To the best of our knowledge, this is the first case of vaginally prolapsed large submucosal uterine fibroid in a woman who delivered vaginally recently. The author recommends that women with known large low situated uterine fibroid should be well observed during the postpartum period to diagnose a vaginally prolapsed uterine fibroid early, so as to prevent fibroid superinfection and obstructive complications.

## Background

Vaginal prolapse of submucosal uterine fibroids is a rare phenomenon during pregnancy, delivery, or puerperium [1, 2]. The prolapse is preceded by strong uterine contractions that are necessary to dilate the cervix before expelling the fibroid. Low situated uterine fibroids measuring 5 cm or more are usually responsible for previa obstacle, with consequently an emergency cesarean section as the only safe mode of delivery [3, 4]. Vaginal prolapse of ≥ 5 cm low situated uterine fibroids during the puerperium in women with recent uncomplicated vaginal delivery might be possible only with an initially intramural fibroid. Thus, large and low situated uterine fibroids may be complicated with previa obstacle during delivery, if not, with delivered myoma in the puerperium with potential obstructive complications. The author reports here on a case of puerperal vaginal prolapse of a large pedunculated submucosal uterine fibroid initially intramural in a woman who delivered vaginally.

## Case presentation

**Fig. 1:**
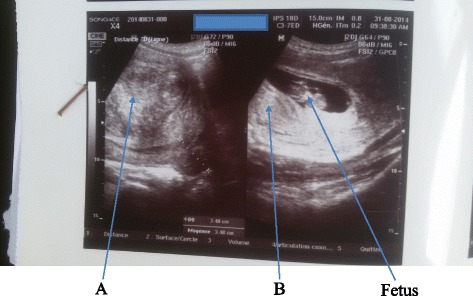
Intramural uterine fibroids diagnosed at 10 weeks. **a** Left lateral intramural uterine fibroid of 15.0 cm diameter (arrow). **b** Posterior intramural uterine fibroid of 7 cm diameter (arrow). The other arrow is pointing to the fetus

A 25-year-old black African woman, gravida 1 para 1, presented on 15 May 2015 with a 5-day history of cramp-like lower abdominal pain radiating to her lower back. This was associated with a foul-smelling hydrorrhea, difficult micturition, and partial urinary retention that occurred 2 days ago. She had a past history of menorrhagia. She had four intramural uterine fibroids measuring 62, 64, 70, and 94 mm diagnosed with standard ultrasound scan in April 2013 during work-up for menorrhagia. She got pregnant in July 2014. A standard ultrasound scan done on 31 August 2014 at 10 weeks’ gestation identified four intramural uterine fibroids, including a left lateral intramural uterine fibroid of 150 mm (biggest) and a posterior intramural uterine fibroid of 70 mm (smallest; Fig. 1). Her pregnancy was well followed up, without any complications. During pregnancy, her uterus had always an irregular shape and a high symphysis-fundal height (20 cm at the 14th week of gestation, 30 cm at the 24th week, 34 cm at the 28th week, and 40 cm at the 38th week). At the 38th week of gestation, two fibroids of 7 cm and 8 cm were palpated on the uterine corpus (the first anteriorly at the right side of the uterine corpus and the second anteriorly on the uterine fundus). At the left lateral side of her uterus, there was also a low situated large fibroid that could not be well palpated and measured. The presentation was cephalic with an unmovable fetal head. The fetal heart tones were normal. She delivered a baby girl, who weighed 3000 g, vaginally on 10 April 2015 at 41 weeks 6 days in the same health facility. A placenta examination revealed retention of parts of fetal membranes. They were removed manually during uterine exploration. During this procedure, no pedunculated submucosal fibroid was found. There were neither maternal nor neonatal complications.

On physical examination at admission, her temperature was 38.5 °C. There was hypogastric tenderness. She had a myomatous uterus with a symphysis-fundal height of 16 cm. There was a foul-smelling vaginal discharge. On speculum and digital vaginal examinations, a mass of approximately 10 cm in diameter was present in her vagina, rendering examination of the cervix difficult.

The diagnosis of an infected prolapsed pedunculated uterine fibroid associated with urinary retention was made. An indwelling urinary catheter was set up, as well as an intravenous drip. Antibiotic therapy was started intravenously with ceftriaxone (1 g twice daily) and metronidazole (500 mg thrice daily). Two days later, under general anesthesia, the almost necrotic fibroid was held with two big toothed forceps and easily twisted off per vaginal route, without any significant bleeding, which relieved her symptoms. On speculum and vaginal examinations, her cervix was normal and 4 cm dilated with the base of the fibroid’s pedicle located in the posterior uterine wall at 3 cm from the external cervical os. The fibroid was sent for pathology, which later confirmed uterine leiomyoma. Five days after fibroid removal, she developed a urethrovaginal fistula due to prolonged urethral obstruction.

## Discussion

Uterine fibroid is the most frequent benign tumor of the female genital tract. The locations can be subserosal, interstitial, or submucosal. Submucosal fibroids, which can be sessile or pedunculated, give more symptoms: menorrhagia; pain due to prolapse process, to red degeneration, or when the stalk of the pedunculated type is twisted; intermenstrual bleeding; and hydrorrhea [5]. Complications of uterine fibroids include anemia (from menorrhagia) and infertility.

During delivery, fibroids may be responsible for placental abruption, abnormal presentation, mechanical dystocia (previa mass) requiring cesarean delivery, and postpartum hemorrhage [6]. None of these complications were observed in our patient.

Other rare complications are vaginal prolapse with compression of the bladder neck or the urethra with resulting urinary retention, as in our case. Urethrovaginal fistula is possible; our patient developed it later [7]. Vaginal prolapse of a fibroid can also occur following uterine artery embolization for uterine fibroids [8]. Uterine fibroids may also induce uterine inversion during prolapse, especially the non-pedunculated submucosal fibroids [9]. Prolapsed pedunculated uterine fibroids are most likely to be infected, especially when they are necrotic, as in our case.

A large submucosal uterine fibroid, low situated in the uterine cavity, is responsible for previa obstacle [4]. The absence of obstacle in our case is explained by the fact that the fibroids were all intramural (as diagnosed with ultrasound scan, regularly palpated abdominally during pregnancy with no pedunculated submucosal fibroid found during uterine exploration), with no obstacle to delivery. In the puerperium, one fibroid became pedunculated submucosal which then prolapsed easily; this phenomenon has been described by some authors [10]. The probable mechanism proposed here is that during the uterine involution process, the intramural fibroid was expelled through the myometrium layer (probably thinner and therefore easily breakable) lining between the endometrium and the intramural fibroid and after through the endometrium. This phenomenon might have been due to uterine contractions, since our patient complained of pelvic pain that started 5 days prior to presentation.

The twisting off of a prolapsed pedunculated submucosal uterine fibroid by means of vaginal route is a simple treatment option [11, 12], which can be done even in a low resource setting. The surgery duration and hospital stay are shorter when compared to laparotomy [13]. Patients should be put on broad spectrum antibiotics [14], as we did for our patient.

## Conclusions

This case report shows that a low situated large intramural uterine fibroid might prolapse vaginally during puerperium. Consequently, women with such fibroids should be well observed during puerperal period to diagnose an eventual vaginal prolapse of the fibroid early, so as to prevent infectious or obstructive complications.
